# Supplementary material for: Identification of photoperiod-induced specific miRNAs in the adrenal glands of Sunite sheep (Ovis aries)
Source: Front Vet Sci. 2022 Jul 22;9:888207. doi: 10.3389/fvets.2022.888207 (PMC9354845; doi:10.3389/fvets.2022.888207)
Supplement: Supplementary file 1 [file Data_Sheet_1.ZIP › Supplementary file/miRDeep2 file.zip!/miRDeep2 file/Novel_Structure/SP42A2/Novel_152.pdf]

Provisional ID	:	NC_019458.2_1458
Score total	:	172.8
Score for star read(s)	:	3.9
Score for read counts	:	165.3
Score for mfe	:	2
Score for randfold	:	1.6
Score for cons. seed	:	
Total read count	:	336
Mature read count	:	314
Loop read count	:	0
Star read count	:	22

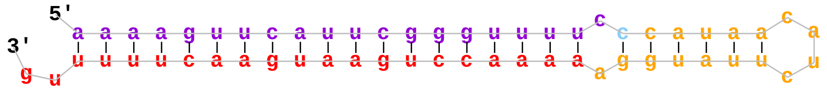

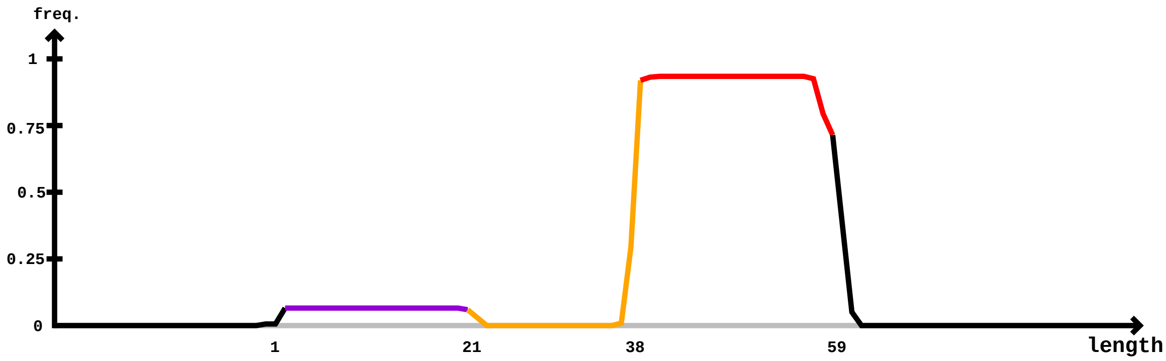

Star **Mature**

```
5'- gaaagaaguauugcgucggcugaaaaguucauucggguuuucccauaacaucuuauggaaaaaccugaaugaacuuuuuggucaacccaauauaaggaggagacaaagaga  -3'  obs
    gaaagaaguauugcgucggcugaaaaguucauucggguuuucccauaacaucuuauggaaaaaccugaaugaacuuuuuggucaacccaauauaaggaggagacaaagaga        exp
    .......((((((.((.((((((((((((((((((((((((.((((((....)))))).)))))))))))))))))))))))).)).)))))))..................  reads  mm  sample
    ....................ugaaaaguucauucggguuuu......................................................................  2   0   seq
    ......................aaaaguucGuucggguuuuc.....................................................................  20  1   seq
    .........................................................gaaaaaccugaaugaacuuuuu................................  3   0   seq
    ..........................................................Uaaaaccugaaugaacuuu..................................  1   1   seq
    ..........................................................aaaaaccAgaaugaacuuuu.................................  13  1   seq
    ..........................................................aaaCaccugaaugaacuuuu.................................  1   1   seq
    ..........................................................aaaaaccugaaugaacuuuu.................................  6   0   seq
    ..........................................................aaaaaccugaaugaacuuuuC................................  1   1   seq
    ..........................................................Uaaaaccugaaugaacuuuuu................................  1   1   seq
    ..........................................................aaaaaccAgaaugaacuuuuu................................  2   1   seq
    ..........................................................aaaaaccugaaGgaacuuuuu................................  6   1   seq
    ..........................................................Gaaaaccugaaugaacuuuuug...............................  1   1   seq
    ..........................................................Uaaaaccugaaugaacuuuuug...............................  1   1   seq
    ..........................................................aaaaaccugaaugaacuuuuug...............................  19  0   seq
    ..........................................................aaaaaccugaaugaacuuGuug...............................  2   1   seq
    ..........................................................aaaaaccAgaaugaacuuuuug...............................  2   1   seq
    ..........................................................aaaaaccugaaCgaacuuuuug...............................  33  1   seq
    ..........................................................aaaaaccugaaugaacuuuuugg..............................  2   0   seq
    ..........................................................aaaaaccugaaugaacuuuuugC..............................  2   1   seq
    ..........................................................aaaaaccugaaGgaacuuuuugg..............................  1   1   seq
    ..........................................................aaaaaccugaaugaacuuuuugU..............................  1   1   seq
    ..........................................................aaaaaUcugaaugaacuuuuuggu.............................  1   1   seq
    ...........................................................aaaaccAgaaugaacuuu..................................  2   1   seq
    ...........................................................aaaaccugaaugaacCuuu.................................  1   1   seq
    ...........................................................aaaaUcugaaugaacuuuu.................................  23  1   seq
    ...........................................................aaaaccAgaaugaacuuuuu................................  9   1   seq
    ...........................................................aaaaccugaaugaacuuuuC................................  1   1   seq
    ...........................................................aaaCccugaaugaacuuuuu................................  1   1   seq
    ...........................................................Gaaaccugaaugaacuuuuu................................  2   1   seq
    ...........................................................aaaaccugaaugaacuuuuug...............................  18  0   seq
    ...........................................................aaaaccugaauAaacuuuuug...............................  1   1   seq
    ...........................................................aaaaccugaaGgaacuuuuug...............................  1   1   seq
    ...........................................................aaaaccAgaaugaacuuuuug...............................  2   1   seq
```

Star Mature

```
gaaagaaguauugcgucggcugaaaaguucauucggguuuucccauaacaucuuauggaaaaaccugaaugaacuuuuuggucaacccaauauaaggaggagacaaagaga
...........................................................aaaaUcugaaugaacuuuuug...............................	57	1	seq
...........................................................aaaaccugaaugaacuuCuugg..............................	1	1	seq
...........................................................aaaaccugaaugaacCuuuugg..............................	1	1	seq
...........................................................Gaaaccugaaugaacuuuuugg..............................	3	1	seq
...........................................................aaaaccugaaugaacuuuuugA..............................	16	1	seq
...........................................................aaaaccugaaugaacuuuuugg..............................	56	0	seq
...........................................................aaaaccugaaugaacuuuuuggG.............................	1	1	seq
...........................................................aaaaUcugaaugaacuuuuuggu.............................	14	1	seq
............................................................aaaccugaaugaacuuuuu................................	1	0	seq
............................................................aaaccugaaugaacuuuuugA..............................	2	1	seq
............................................................aaaccugaaugaacuuuuugUu.............................	1	1	seq
.............................................................aaccugaaugaaAuuuuug...............................	1	1	seq
```
